# Testing Alpha-1 Antitrypsin Deficiency in Black Populations

**DOI:** 10.3390/arm92010001

**Published:** 2023-12-19

**Authors:** Pascale Lafortune, Kanza Zahid, Magdalena Ploszaj, Emilio Awadalla, Tomás P. Carroll, Patrick Geraghty

**Affiliations:** 1 Department of Medicine, State University of New York Downstate Medical Center, Brooklyn, NY 11203, USA; pascale.lafortune@downstate.edu (P.L.); kanza.s.zahid@gmail.com (K.Z.); magdalena.ploszaj@nyulangone.org (M.P.); emilio.awadalla@bath.edu (E.A.); 2 Irish Centre for Genetic Lung Disease, Royal College of Surgeons in Ireland, D02 YN77 Dublin, Ireland; 3 Alpha-1 Foundation Ireland, Royal College of Surgeons in Ireland, D02 YN77 Dublin, Ireland

**Keywords:** alpha-1 antitrypsin deficiency, chronic obstructive pulmonary disease, Black populations, genetic screening

## Abstract

**Highlights:**

What are the main findings?

- Alpha-1 antitrypsin deficiency is extensively studied in populations of European ancestry but other ethnic populations also carry *SERPINA1* mutations that may be harmful to these populations.
- The majority of studies undertaken in non-European populations screen for *SERPINA1* mutations in small subject numbers and not from the general population.

What is the implication of the main finding?

- Insufficient alpha-1 antitrypsin deficiency testing is performed in Black populations that already experience poor health outcomes.
- Diagnosis of severe *SERPINA1* mutations and counseling assists patients in their health education and a diagnosis of alpha-1 antitrypsin deficiency is a stronger motivator to quit smoking, improves the frequency of regular health checks, and lung and liver scans in patients.

**Abstract:**

Alpha-1 antitrypsin (AAT) deficiency (AATD) is an under-recognized hereditary disorder and a significant cause of chronic obstructive pulmonary disease (COPD), a disease that contributes to global mortality. AAT is encoded by the *SERPINA1* gene, and severe mutation variants of this gene increase the risk of developing COPD. AATD is more frequently screened for in non-Hispanic White populations. However, AATD is also observed in other ethnic groups and very few studies have documented the mutation frequency in these other ethnic populations. Here, we review the current literature on AATD and allele frequency primarily in Black populations and discuss the possible clinical outcomes of low screening rates in a population that experiences poor health outcomes and whether the low frequency of AATD is related to a lack of screening in this population or a truly low frequency of mutations causing AATD. This review also outlines the harmful *SERPINA1* variants, the current epidemiology knowledge of AATD, health inequity in Black populations, AATD prevalence in Black populations, the clinical implications of low screening of AATD in this population, and the possible dangers of not diagnosing or treating AATD.

## 1. Introduction

Black populations experience significant health inequities that lead to poor outcomes, such as higher rates of chronic diseases, maternal and infant mortality, infectious diseases, mental health issues, and lower life expectancy [1]. Some of these outcomes can be addressed by improving access to healthcare, addressing social determinants of health, and tackling discrimination and bias in healthcare and within our society. However, this excess burden is well documented and is not completely explained by socioeconomic status or access to care [2]. With the recent COVID-19 pandemic, one study reported that 27% of associated deaths within the USA were Black patients, while Black patients account for only 12.5% of the population in the USA [3]. In 2020, chronic liver disease was the ninth leading cause of death for African Americans, ages 45–64 years old [4], and in a single-center study, African American patients with antineutrophilic cytoplasmic antibody (ANCA)-associated vasculitis were diagnosed at a younger age than Caucasian patients [5]. Studies looking at ethnic differences in COPD diagnosis also demonstrate the underdiagnosis of the disease in Black populations [6]. In 2003, the American Thoracic Society (ATS) and European Respiratory Society (ERS) recommended that all individuals with a diagnosis of chronic obstructive pulmonary disease (COPD), refractory asthma, unexplained chronic liver disease, or panniculitis, irrespective of age or ethnicity, should be tested for alpha-1 antitrypsin deficiency (AATD) [7]. Since AAT is an acute phase protein, it is recommended to perform iso-electric focusing testing for the common alleles of AATD in these individuals. While AATD continues to be underdiagnosed in all populations, the frequency and nature of harmful serine protein inhibitor-A1 (*SERPINA1* gene) mutations that occur in Black populations are unclear. Since Black populations experience significant health inequities, one could expect similar disparities in AATD diagnosis and treatment. In this mini-review, we want to outline the current literature on AATD and *SERPINA1* mutation frequency in Black populations and discuss the consequences of low screening rates in a population that already experiences disproportionately poor health outcomes compared to other ethnic groups. Equally, we discuss whether the reported low prevalence of AATD is related to a lack of screening in this population or simply a low frequency of *SERPINA1* mutations causing AATD.

## 2. AATD: A Historical and Biological Perspective

AAT was first characterized as a protease inhibitor, with loss in this activity associated with lung disease by Swedish researchers in 1963 [8]. AAT is a 52 kDa glycoprotein produced mainly by hepatocytes and secreted into the blood. AAT primarily inhibits neutrophil elastase, and in the absence of AAT, unregulated active proteases cleave the structural proteins of the lungs. AAT also has a plethora of anti-inflammatory and immune-modulatory properties. AAT deficiency is the most common genetic cause of COPD. Without sufficient concentrations of biologically active AAT, tissue destruction and airspace enlargement can occur, leading to progressive emphysema. This process is accelerated by exposure to cigarette smoke or other environmental factors. COPD development is common in AATD, especially in combination with cigarette smoke exposure. AATD patients with a smoking history typically present with emphysema on a chest computed tomography and with obstruction determined by spirometry. Chronic bronchitis or asthma is also observed but less frequently [9]. However, AATD subjects who do not smoke tend to get radiographic emphysema after 60 years old [10]. Asthma may be more prevalent in AATD individuals, as wheezing and dyspnea are some of the first pulmonary symptoms in AATD [11]. Bronchiectasis is also increased in AATD [12], and this is associated with atypical mycobacterial infection [13]. Therefore, AAT testing is recommended for subjects with emphysema, COPD, bronchiectasis, chronic bronchitis, and asthma where spirometry fails to return to normal upon the treatment of asthma [7]. Approximately 2–3% of patients diagnosed with COPD will be AATD [14]. AATD is also a significant cause of liver disease through the polymerization and accumulation of misfolded Z AAT protein within hepatocytes and is a common cause of liver transplantation. Since lung disease in AATD is almost indistinguishable from nonhereditary lung disease, AATD is an under-recognized hereditary disorder and screening typically occurs after disease establishment. AATD is often suspected only following the diagnosis of early-onset obstructive lung disease in individuals with minimal or no cigarette consumption or panacinar emphysema affecting mainly the lower lobes.

AAT is encoded by the *SERPINA1* gene. The most common mutation known to cause severe AAT deficiency is Z (p.Glu342Lys, rs28929474). Individuals homozygous or heterozygous for the Z mutation are at increased risk of developing COPD, although heterozygotes require a second insult such as smoking before developing COPD [4,15,16,17]. Classically, severe AATD is more frequently screened for in non-Hispanic White populations. Therefore, AATD is primarily documented in Northern, Western, and Central Europe. However, AATD is also observed in other ethnic groups [18]. This is important as COPD is the fourth leading cause of death in the United States of America, with over 16 million (6.6%) people reporting a diagnosis of COPD [19]. In 2019, COPD was the third leading cause of death worldwide, causing 3.23 million deaths [20]. Self-reported COPD is estimated to be 6.1% in Black populations [21]. Though COPD is typically diagnosed in patients who are current and or former smokers, one in four people who are nonsmokers develop COPD [22]. In addition to environmental factors, individuals who have a genetic predisposition, such as AATD, can develop COPD. A recent study demonstrated that the primary factors for favoring AAT testing were whether the patient was of the White race and had concomitant COPD and liver disease [23]. This same study also observed that increasing age, being non-White, current tobacco use, and being a male with COPD reduced the odds of AAT testing being performed [23]. Therefore, there is bias in performing AAT testing and screening in certain populations.

## 3. AATD Mutations

*SERPINA1* is a pleiomorphic gene with alleles inherited in an autosomal co-dominant fashion. The most frequent clinically significant alleles are Z and S, with M being the normal/non-mutated allele. It is important to note that the S allele (p.Glu264Val, rs17580) is less polymerogenic and causes mild serum deficiency. Thus, the SZ genotype results in a phenotype similar to the MZ phenotype and is deemed less severe than ZZ [24]. There are a few published unbiased studies that address the allele frequencies in the general population. Most studies perform AAT testing on cohorts with a high number of COPD subjects or other subjects with already diagnosed pulmonary diseases, which do not reflect the true numbers in the general population. In a recent study from the Canary Islands (Spain), the estimated frequency of S and Z alleles in the general population was 8.2% and 2.1%, respectively [25]. An Irish study found an estimated prevalence of 1/25 (4%) for the Z allele and 1/10 (10%) for the S allele in DNA collected from 1100 individuals randomly sampled from the general population [26]. In a large genetic testing study, the allele frequency for the Z and S variants among 195,014 study participants was 6.5% and 15.1%, respectively. Notably, this cohort included 1443 African Americans [27]. Unfortunately, allele frequencies in the African American group were not reported. Finally, the Genome Aggregation Database (gnomAD), which is a shared aggregate exome and genome sequencing database from a variety of large-scale sequencing projects [28], is a useful tool for looking at *SERPINA1* variants in multiple ethnic populations, including African and African American populations. These datasets may represent a better overall frequency for *SERPINA1* variants in multiple populations.

Recent advances in sequencing have identified large numbers of harmful new *SERPINA1* variants, with over 200 identified to date [29]. There are many rare *SERPINA1* variants that could be population specific [30] but require further investigation. Table 1 outlines some of these harmful *SERPINA1* variants. All these variants are predicted to be observed in Black populations and require further study. A recent study that compiled a comprehensive database of *SERPINA1* coding mutations reported that 2.59% of an African cohort carry harmful SERPINA1 mutations [31].

Severe AATD affects about 1 in 1500 to 3500 individuals with European ancestry. While several studies do show lower frequencies in other ethnic groups, the Z and S alleles are documented to be detected in countries in the Caribbean, North and South America, Asia, and Africa [39]. Historically, detection has focused on ZZ AATD, but it is now accepted that Z heterozygotes are also at risk of COPD. A study in Ireland found that MZ smokers were at a higher risk of developing COPD when compared to MM siblings who smoked [15]. The finding of increased risk and severity of COPD in Z heterozygotes has been replicated in larger, multi-ethnic cohorts [4,16,17]. One of these studies showed that African American MZ subjects had lower lung function, observed with low FEV_1_ percent predicted and FEV_1_/FVC compared to African American MM subjects [16]. It is clear that MZ smokers are at risk for lung function changes in both White and Black populations. 

### Regulation of the SERPINA1 Gene

The regulation of the *SERPINA1* gene is quite complex. An epigenome-wide association study (EWAS) in 2012 was performed on peripheral blood mononuclear cells from adults who were smokers and suggested a positive correlation between hypomethylation at two CpGs in the *SERPINA1* gene promoter and COPD risk [40]. However, another study performed on samples from smoke-exposed children and adults observed no correlation between *SERPINA1* gene methylation and lung function [41]. A recent study demonstrated that the *SERPINA1* gene promoter is differentially methylated in peripheral blood mononuclear cells from healthy subjects [42]. However, further studies are needed to assess the direct link between AAT circulating levels and *SERPINA1* promoter methylation in blood cells. There are 11 known *SERPINA* mRNA isoforms, which are generated through alternative splicing involving the 5′-UTR of the pre-mRNA [43,44]. Mutations in *SERPINA1* 5′-UTR non-coding regions can lead to altered translation, as observed in a large-scale clinical study looking at AAT serum levels in patients [17]. One of these 5′-UTR (NM_000295.4) can reduce AAT translation [45]. This may be another means of observing altered AATD levels in different populations.

## 4. Health Outcomes in Black Populations

The Black population in the United States of America has worse health outcomes in comparison to other ethnic groups [1]. A significant number of physicians are unaware of the current guidelines for screening for AATD in patients and may not be aware of possible treatment available for this form of COPD [46]. Therefore, this may further lead to a reduced urgency of making a diagnosis of AATD. A recent whole genome sequence (WGS) study examined gene variants and lung function and COPD and identified two common variant signals unique to lung function in African Americans [47]. There is likely heterogeneity in genetic effects when investigating race/ethnicity and lung function. Therefore, we cannot presume genetic variants associated with lung function identified by GWAS may be applicable to all populations. Unique variant signaling associated with certain ethnic populations requires further investigation.

### AATD Prevalence in Black Populations

Several studies estimate the global frequency of AATD and *SERPINA1* variants in different populations worldwide [48]. Studies looking at 94 countries encompassing 75% of the global population estimated that 173,430 individuals possess the ZZ genotype and 1,011,069 the SZ genotype [49]. Importantly, a recent study predicted that more than 35 million people in 74 countries possess the MZ genotype [50].

A study conducted comparing Black and White populations with emphysema demonstrated that Black subjects had a similar degree of lung impairment compared with Whites but developed emphysema younger despite smoking less [51]. As seen in Table 2, consisting of studies reporting Black population screening, only a small number of studies report actual numbers detected during screening, and AAT mutations are detected in this population. Some studies do state that non-White subjects were tested but do not provide screening data based on race [52,53]. Estimated frequencies exist in Z and S alleles in Caribbean and African countries; the most significant, in Cuba, Dominican Republic, Puerto Rico, Nigeria, Somalia, Angola, and Namibia, are reported [50]. It is important to note that many estimates are based on studies from the 1970s and 1980s in small cohorts and not necessarily the general population. This could warrant further AAT screening in larger Black cohorts with newer diagnostic techniques.

Finally, one must also consider that spirometry reference values differ by race/ethnicity, which could result in the underestimation of COPD in Black populations [66]. Applying spirometry reference equations used for Caucasian populations may produce normal lung function values (% predicted) in Black populations [67]. Equally, the smoking habits between populations differ, with cigarette products containing menthol and other flavorings frequently being targeted at Black smokers and vaping device users [68]. Equally, there is some evidence to suggest that racial/ethnic minority populations and younger smokers find it harder to quit menthol versus nonmenthol cigarettes [69].

## 5. Screening and Diagnosis of AATD

A new ERS statement on AATD outlines an extended algorithm for family screening of individuals diagnosed with severe AATD, including their close relatives and spouses [70]. Even with guidelines in place, there are still significant delays between the appearance of symptoms and the correct diagnosis of AATD. The recommended approach to testing for AATD is to first measure plasma or serum AAT levels. However, it should be noted that AAT levels have weak intra-individual reproducibility due to the acute phase nature of AAT [71]. This could result in a missed AATD diagnosis. Thus, CRP should be ordered in combination with AAT to rule out falsely elevated AAT levels due to illness or inflammation. If AAT is abnormally low, further testing should be performed by either AAT phenotyping with isoelectric focusing or AAT mutation-specific genotyping [72]. The gold standard to detect AATD is DNA sequencing, especially for rarer variants extended molecular techniques are required, such as whole exome sequencing [73]. A recent study in Greece observed several new rare variants by sequencing and these variants appear to be pathogenic as they were detected in patients with early emphysema and lower than normal AAT levels [74]. Non-coding DNA may be an important area to be assessed [75]. For example, the integrative deep sequencing of *SERPINA1* identified a 5′ untranslated region insertion (rs568223361) in African Americans that is associated with lower AAT levels and an increased risk of small airway disease [17]. Commercially available direct-to-consumer genetic tests have also allowed people to explore the possibilities of genetic screening. However, one needs to be cautious with over-the-counter genetic testing as this results in the customer paying for the service, and these tests are not federally regulated in regard to quality control and provide no counseling service upon the identification of possible genetic diseases. A recent study using data from the U.S. Bronchiectasis Research Registry found that non-Hispanic Black patients were tested less frequently for AATD compared to other groups [76]. This was not the case for screening for cystic fibrosis, immunoglobulin deficiency, and mycobacteria [76].

### Clinical Implications of Low Screening Rates

Early recognition of AATD is critical as it permits interventions, including education (e.g., smoking cessation and avoidance), genetic counseling, testing family members, and specific treatment options. The cessation of smoking is strongly recommended following diagnosis of AATD. Equally, the initiation of early additional clinical interventions is paramount, such as bronchodilator and inhaler therapies, pulmonary rehabilitation, and lung volume reduction or lung transplantation in severe cases [70]. Delayed diagnosis is also associated with a negative psychosocial impact, which could be mitigated [77]. For each additional year of diagnostic delay in AATD, FEV1% predicted decreases by 0.3%, the St. George Respiratory Questionnaire total score increases by 1.6 points, and the COPD Assessment Test score increases by 0.7 points [77]. Equally, determining the precise AATD genotype is a major indicator of disease risk, with the ZZ and rare ZZ-equivalent genotypes associated with a higher risk of COPD and liver disease.

Without neonatal screening, or at the very least systematic targeted screening for those with obstructive lung disease and unexplained liver disease, the true prevalence of AATD will remain undetermined, and the disease will continue to be underdiagnosed. Currently, the ATS/ERS does not recommend screening neonates or adolescents [7], but this would facilitate early education and intervention.

## 6. Benefits of Diagnosing AATD

A correct diagnosis of AATD provides patients and physicians with a variety of management and treatment options. These include consultation with a genetic counselor to discuss the diagnosis, treatment options, lifestyle changes, and the screening of other family members. Diagnosis is particularly important as it permits the screening of relatives, as they need to consider AATD in their clinical history and maintain surveillance and risk-reduction for liver and lung diseases (such as alcohol consumption and smoke cessation). A recent study demonstrated that the greater the severity of the AATD genotype, the lower the smoking rates among ever-smokers, with a diagnosis of AATD shown to be a stronger motivator to quit smoking than a diagnosis of COPD [78]. Diagnosed AATD individuals also should consider vaccination strategies, undergo regular health checks, and lung and liver scans.

In those with emphysema caused by severe AATD, weekly intravenous (IV) infusions of plasma-purified AAT, known as augmentation therapy, are an effective treatment option. A recent trial demonstrated that patients with emphysema caused by severe AATD treated with AAT augmentation therapy showed a slower radiologic progression of the disease as compared to placebo [79]. In addition, the discontinuation of AAT augmentation therapy in an Irish ZZ population prompted a deterioration of lung disease, including increased exacerbations following the abrupt cessation of treatment [80].

It is important to note that the costs of AAT augmentation therapy are high within the USA and throughout the world. One study published in 2018 found the total medical cost of patients on augmentation therapy to be USD 127,000, while the cost of patients not on therapy was USD 15,874 annually for insurance companies [81]. Disparities already exist in the diagnosis of AATD, and the high cost of treatment may have an impact on populations unable to access this expensive therapy.

## 7. Conclusions

**Figure 1:**
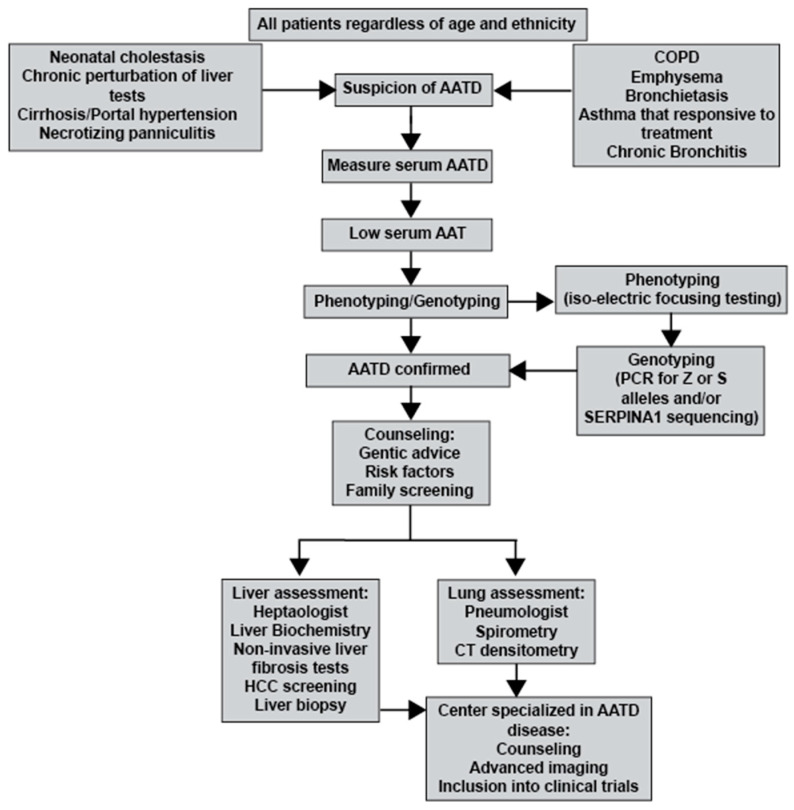
Flowchart for the testing of AATD and management/treatment options.

AATD is substantially underdiagnosed, with an estimated 10% of predicted severe cases of AATD diagnosed. Most of the available data regarding the mutation frequency of SERPINA1 variants in Black populations are estimates. A small number of studies provide actual data on AATD in Black populations, but these cohorts are very small. The increasing frequency of transnational or multiracial relationships may also affect the presence of AAT alleles in these populations. Comparing the prior clinical epidemiological studies from earlier studies to large existing datasets (such as the gnomAD database) would likely increase our knowledge of the true frequencies of *SERPINA1* variants and AATD in Black populations. The recent improvement in diagnosis and treatment further emphasizes the importance of identifying AATD in Black populations, thereby providing this neglected population with appropriate and more effective medical care and treatment options. See Figure 1 for possible AATD testing, management, and treatment strategies for subjects with a suspicion of AATD independent of their age or ethnicity.

## Figures and Tables

**Table 1:** Harmful *SERPINA1* variants frequently identified in AATD screening programs.

SERPINA1 Variant	Molecular Basis	SNP Number	Cellular Effect	Disease Association	Reference	Observed in Non-European Populations?
S	p.Glu264Val	rs17580	Polymerization, impaired secretion, reduced antiprotease activity	Lung & liver if inherited with other severe AATD variant(s) (e.g., Z)	Lomas et al., 1999 [32]	Yes
Z	p.Glu342Lys	rs28929474	Polymerization, impaired secretion, reduced antiprotease activity	Lung & liver	Laurell & Eriksson, 1963 [33]	Yes
I	p.Arg39Cys	rs28931570	Polymerization, impaired secretion, reduced antiprotease activity	Lung & liver	Lomas et al., 1999 [32]	Yes
F	p.Arg223Cys	rs28929470	Reduced antiprotease activity	Lung	Fagerhol & Tenfjord, 1968 [34]	Yes
M_malton_	p.Phe52del	rs775982338	Polymerization, impaired secretion, reduced antiprotease activity	Lung and liver	Curiel et al., 1989 [35]	Yes
M_wurzburg_	p.Pro369Ser	rs61761869	Intracellular accumulation & polymerization	Lung and liver	Poller et al., 1999 [36]	Yes
M_heerlen_	p.Pro369Leu	rs199422209	Block in production	Lung	Poller et al., 1999 [36]	Yes
S_iiyama_	p.Ser53Phe	rs55819880	Polymerization	Lung and liver	Lomas et al., 1993 [37]	Yes
Null (Q0)	Premature termination codon	N/A	Family of mutations that produce no detectable AAT	Lung	Talamo et al., 1973 [38]	Yes

Single nucleotide polymorphism (SNP), not available (N/A).

**Table 2:** Studies reporting AATD screening in Black populations.

Study	Location	Study Population Number (N)	AAT Genotype					
			MM	MS	MZ	SS	SZ	ZZ
Spınola et al. [54]	Cape Verde Islands	202	191	7	1	3	0	0
Foreman et al. [16]	USA; COPDGene cohort ^a^	2803	2731	49	22	0	1	0
Miskoff et al. [55]	Neptune Township, New Jersey, USA	18	16	1	1	0	0	0
Ashenhurst et al. [27]	23andMe customers	1443	N/A	N/A	N/A	N/A	N/A	N/A
Ortega et al. [17]	USA; SPIROMICS cohort	385 ^b^	N/A	N/A	N/A	N/A	N/A	N/A
Denden et al. [56]	Tunisia; obstructive lung disease cohort	120	119	0	1	0	0	0
Webb et al. [57]	Rochester, Monroe County, NY, USA	53 ^c^	N/A	N/A	N/A	N/A	N/A	N/A
Young et al. [58]	Washington, DC, USA	94 ^d^	N/A	N/A	2	N/A	1	1
Pierce et al. [59]	St. Louis, MO, USA	204	196	4	2	N/A	N/A	N/A
Massi et al. [60]	Mogadishu, Somalia	347	333	9	1	0	1	3
Vandeville et al. [61]	Zaire	132 ^e^	124	0	0	0	0	0
Welch et al. [62]	Gambia, West Africa	701 ^f^	700	0	0	0	0	0
Pascali et al. [63]	Congo, West Africa	278	243	35	0	0	0	0
Chaabani et al. [64]	Tunisia	310	260	50	0	0	0	0
Giacopuzzi et al. [31]	Multiple public databases	5203 ^g^	N/A	81	35	N/A	116	N/A
Lieberman et al. [65]	High school students from Long Beach, CA, USA	186 ^h^	182	3	0	0	0	0

^a^ Denotes studies with smokers with and without COPD only, i.e., not the general population. Data only for MM and MZ groups; ^b^ Study looked at missense and frameshift exonic *SERPINA1* variants and identified four variants unique to African Americans. Exact numbers not outlined in the manuscript for African American data breakdown; ^c^ Study reported that 3 of the 53 African Americans were not MM. However, no additional information given; ^d^ Study consisted of 559 African American ambulatory and hospitalized patients with high-risk cardiopulmonary disorders. However, AAT screening is only outlined in 94 COPD-positive patients. Other data are combined with data from 115 control Caucasian patients; ^e^ Other mutation phenotypes detected: LM = 7, MV = 1; ^f^ PiGAM variant detected, n = 1; ^g^ 2.59% of African cohort carry harmful *SERPINA1* mutations, ^h^ One subject was EM phenotype. N/A, not available or data not detailed in manuscript.

## Data Availability

Not applicable.

## Abbreviations

Alpha-1 antitrypsin, AAT; Alpha-1 antitrypsin deficiency, AATD; chronic obstructive pulmonary disease, COPD; American Thoracic Society, ATS; European Respiratory Society, ERS; Antineutrophilic cytoplasmic antibody, ANCA; whole genome sequence, WGS.
